# Combination of Heat Shock and Enhanced Thermal Regime to Control the Growth of a Persistent *Legionella pneumophila* Strain

**DOI:** 10.3390/pathogens5020035

**Published:** 2016-04-15

**Authors:** Emilie Bédard, Inès Boppe, Serge Kouamé, Philippe Martin, Linda Pinsonneault, Louis Valiquette, Jules Racine, Michèle Prévost

**Affiliations:** 1 Department of Civil Engineering, Polytechnique Montréal, Montréal, QC H3T 1J4, Canada; ines.boppe@polymtl.ca (I.B.); michele.prevost@polymtl.ca (M.P.); 2 CIUSSSE—Centre Hospitalier Universitaire de Sherbrooke, Sherbrooke, QC J1G 2E8, Canada; skouame.chus@ssss.gouv.qc.ca (S.K.); linda.pinsonneault@usherbrooke.ca (L.P.); jracine.chus@ssss.gouv.qc.ca (J.R.); 3 Department of Microbiology and Infectious Diseases, Université de Sherbrooke, QC J1K 2R1, Canada; Philippe.Martin@USherbrooke.ca (P.M.); Louis.Valiquette@USherbrooke.ca (L.V.)

**Keywords:** legionellosis, sanitary hot water, temperature diagnostic, *Legionella pneumophila*, culture, quantitative polymerase chain reaction, copper concentration, hospital premise plumbing

## Abstract

Following nosocomial cases of *Legionella pneumophila*, the investigation of a hot water system revealed that 81.5% of sampled taps were positive for *L. pneumophila*, despite the presence of protective levels of copper in the water. A significant reduction of *L. pneumophila* counts was observed by culture after heat shock disinfection. The following corrective measures were implemented to control *L. pneumophila*: increasing the hot water temperature (55 to 60 °C), flushing taps weekly with hot water, removing excess lengths of piping and maintaining a water temperature of 55 °C throughout the system. A gradual reduction in *L. pneumophila* counts was observed using the culture method and qPCR in the 18 months after implementation of the corrective measures. However, low level contamination was retained in areas with hydraulic deficiencies, highlighting the importance of maintaining a good thermal regime at all points within the system to control the population of *L. pneumophila*.

## 1. Introduction

*Legionella pneumophila* is an opportunistic pathogen responsible for a significant number of nosocomial infections. *L. pneumophila* outbreaks reported in hospitals have predominantly been associated with the hot water systems (HWS) within hospital premises [1,2,3,4]. The presence of *L. pneumophila* in the HWS of healthcare facilities is well documented, with reports of 10% to 50% positive in water samples from taps and showers [5,6,7,8,9]. Hospital-acquired legionellosis can result in prolonged hospitalization and elevated mortality rates [10]. Although an infectious dose has not been established, a guidance document published by the Health and Safety Executive (HSE) provides recommendations regarding the actions to take in the presence of *Legionella* in the water system at levels above 100 CFU/L [11]. In hospital settings, a dose as low as 10 CFU/mL may be sufficient to cause an infection [12]. Several European countries, such as Austria, France, Germany, Italy, the United Kingdom and Switzerland, have adopted guidelines or regulations that specify an alert level and an action level [11,13,14,15,16,17,18]. In general, the alert level is set between 100 and 1000 CFU/L and prescribes further investigation to define if there is a system contamination or if the problem is localized. Corrective actions are recommended for identified issues. There is a consensus to set the action level at 10,000 CFU/L, a high level of contamination at which corrective action should be taken immediately. Alert and action levels for results obtained by quantitative polymerase chain reaction (qPCR) were suggested at 4000 genomic units (GU)/L and 40,000 GU/L, respectively [19].

The control of *Legionella* to reduce the risk of exposure in healthcare facilities has been addressed in multiple guidance documents [20]. Approaches to control *L. pneumophila* in hot water distribution systems vary considerably, but all guides include objectives or obligations for ensuring optimal operating temperatures at critical points in the distribution systems. A key control measure is to maintain elevated water temperatures and to ensure optimal water circulation through a recirculation loop. Most regulations and guidelines suggest a temperature of 60 °C at the water heater outlet and a minimal water temperature of 55 °C across the network. Recent guidelines also highlight the importance of efficient hydraulics with recommended flow-rates of 0.2 m/s, to ensure homogeneous temperature and biocidal control throughout the hot water system [21]. Previously, diagnostic and assessment tools to evaluate the risk of *L. pneumophila* proliferation were proposed, based on the water temperature at critical control points (the hot water heater outlet, the point-of-use (after 1 to 2 min of water flow) and the recirculation loop) [20].

Ideally, systems should be hydraulically optimized and temperatures maintained throughout the system [11,22]. However, if this cannot be achieved or if sampling results indicate microbial contamination, disinfection should be applied. There are several water disinfection methods used to control *Legionella* in hot water distribution systems [23], including heat shock, implementation of a thermal regime and copper-silver ionization. However, no disinfection method has proven wholly effective, especially against the biofilm established within the pipes. The development of resistance following multiple heat shocks in a hospital hot water distribution system has been documented. The implementation of thermal regimes has successfully been used to reduce the percentage of taps positive for *Legionella* [2,24,25]. Taps that remained positive despite the implementation of a thermal regime were associated with faulty hydraulics and lower water temperatures at the tap [24,25]. Indeed, *Legionella* was reduced in systems where hot water temperatures at the tap exceeded 55 °C within 1 to 2 min of water flow [5,6,20,26]*.* A review summarizes the successful use of copper silver ionization in several studies reported to reduce levels of *L. pneumophila* in hospital hot water distribution systems [23]. However, there are also reports of unreliable eradication of *L. pneumophila* following copper silver-ionization treatment, which resulted in cases of legionellosis [3,25].

Despite the application of disinfectants or the establishment of thermal regimes, episodes of periodic interruption in the application of the control method can promote system contamination. Monitoring of the microbiological quality of water is needed to determine the efficacy of treatment. However, disinfectants and other environmental stressors are known to induce a viable, but not culturable (VBNC) or intra-amoeba state in *L. pneumophila*, without causing cell death [27,28]. Culture techniques could be an unreliable indicator of the population size of *L. pneumophila* under such conditions. System-wide failure or local hydraulic conditions at the tap (loss of disinfectant or temperature) represent situations where VBNC cells could regain culturability and proliferate, especially in the presence of biofilm. Molecular detection methods, such as quantitative polymerase chain reaction, provide interesting means of monitoring the impact on *L. pneumophila* system contamination following the implementation of new control measures. This method has been used previously for the detection of *L. pneumophila* in water distribution systems [19,29,30].

The present study was conducted at an academic tertiary care hospital where two cases of nosocomial legionellosis were reported in August 2014. An environmental investigation was conducted, and the hot water system was identified as the source of infection. Isolated environmental and clinical strains revealed the same pulsed-field gel electrophoresis (PFGE) patterns and were also from the same sequence-based type (sequence type-1427). At the time of outbreak, the temperature set point at the water heater outlet was 55 °C, and a temperature of 50 °C was maintained in the main recirculation pipe. A copper-silver ionization system was present in the studied system.

The objectives of this study were to: (1) evaluate short-term *L. pneumophila* survival after thermal disinfection; (2) identify areas at risk due to *L. pneumophila* proliferation, using the temperature diagnostic method; and (3) assess the long-term effects of ongoing thermal controls and hydraulic improvements, at key points-of-use, over an 18-month period, using culture-based and qPCR monitoring approaches.

## 2. Results and Discussion

The HWS feeding the hospital wing where the outbreak originated (System A) was investigated and monitored closely in the year following the outbreak. A second, independently-operated HWS (System B), feeding an adjacent building wing with no reported cases of legionellosis, was also investigated. Following a full decontamination of each HWS by thermal disinfection, a temperature diagnostic was initiated to identify and prioritize risk areas in each building wing. Corrective measures were put in place, and long-term effects were monitored through periodical sampling of *L. pneumophila*, at various points of the HWS.

### 2.1. Effect of Thermal Disinfection on Legionella pneumophila

A thorough thermal disinfection was conducted, involving the circulation of water at temperatures above 70 °C, for at least 30 min, in all primary and subordinate loops. Primary and subordinate flow and return loops were previously defined [20]. Each point-of-use was flushed for a minimum of 7 min at a temperature ≥70 °C. The impact of the thermal disinfection was evaluated by sampling 250 mL of first flush hot water, at different points-of-use. Results suggest that thermal disinfection was successful at reducing levels of culturable *L. pneumophila* in System A, but no change was observed in the levels recovered from System B (Table 1).

System B had a larger number of distal points relative to System A. As such, in System B, thermal disinfection was conducted in two separate interventions, one week apart, whereas in System A, a single intervention was required (see Section 3.2). This variation in protocol may have contributed to the reduced effectiveness of thermal disinfection seen in System B. The recirculating loop of the thermally-disinfected section in System B was isolated to prevent recontamination of the piping during the period between interventions. However, in some sectors of System B, sub-optimal recirculation was observed, with some areas requiring up to 20 min to reach hot water system temperatures of 70 °C. It is possible that in these sectors, despite the thermal disinfection procedure, the inability to maintain high temperatures over a long enough period enabled the regrowth of *L. pneumophila*, which reseeded the system through the recirculating loop [11]. Another factor to consider is the initial level of contamination. In System A, initial levels were 10× higher than in System B. Furthermore, the relative effectiveness of the thermal disinfection is based on culture results with a detection limit of 1000 CFU/L. This value is unacceptably high for accurately concluding whether *L. pneumophila* levels proposed in international recommendations and regulations (Table 1) were met in this study. As a result, the sampling protocol and laboratory were changed for the subsequent analysis, as presented in Section 3.4.

### 2.2. Hot Water System Temperature Diagnostic

**Figure 1:**
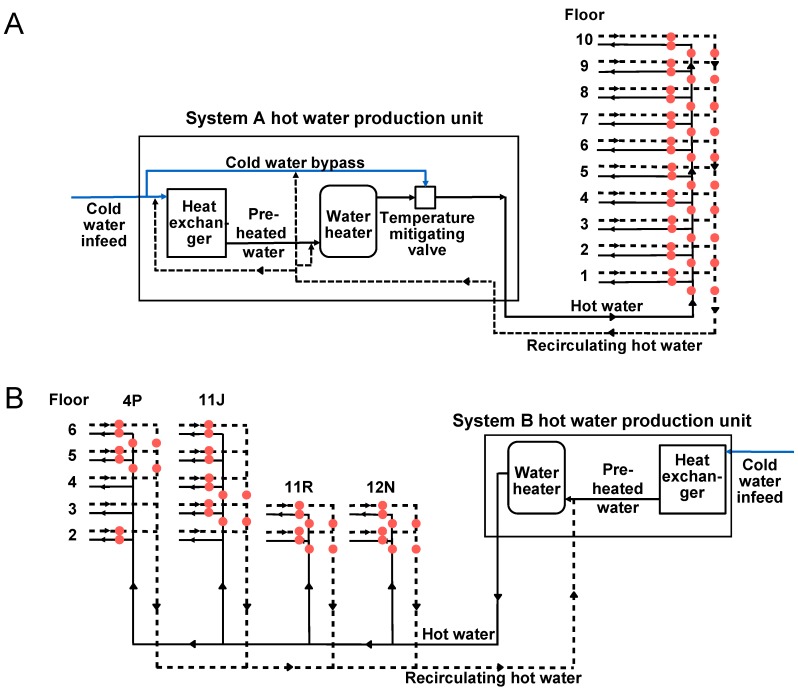
Hot water system schematic for System A (**A**) and System B (**B**) where red circles represent the temperature probe location.

The hot water systems were investigated using the previously-proposed diagnostic flow chart [20]. A detailed characterization of both studied systems was performed, followed by principal and subordinate flow loop temperature monitoring. Figure 1 presents a schematic of the studied systems. In both systems, the water from the principal recirculation loop flows into the vertical secondary distribution pipes, which bring water to each floor through a subordinate horizontal loop. System A only has one vertical pipe delivering water to each subordinate horizontal flow loop, whereas four vertical distribution pipes are present in System B (Figure 1).

#### 2.2.1. Principal Recirculation Loop

The mean hot water temperature coming out of the hot water production unit in System A was 60.3 ± 0.28 °C compared to 60.0 ± 0.1 °C in System B. In System A, the water is heated in a flash heat exchanger to a mean temperature of 70 °C and mitigated to 60 °C by adding recirculating water or make-up cold water as needed (Figure 1a). This results in very stable temperatures feeding into HWS A (Figure S1). On the other hand, although the standard deviation is comparable in both systems, detailed analysis of continuous temperature monitoring results reveals a more important variation in periods of high usage for System B, such as during week days (Figure S1). In System B, the water is heated in a flash heat exchanger to a mean temperature of 60 °C and fed directly into the system without the presence of a mitigating valve. Despite the absence of a mitigating valve, results show the ability of System B to promptly react to temperature fluctuations associated with increased use of hot water for showering and bathing. Principal loop recirculation temperatures, prior to re-entering the hot water production unit, were 54.7 ± 0.29 °C in System A and 56.2 ± 0.3 °C in System B. The heat loss between the water heater and the return loop is within recommended values of 5 °C and indicates a good overall circulation within the systems. These data confirm the capacity of the water heaters in both systems to provide the required temperatures, more than 90% of the time. However, this apparent compliance with prescribed temperatures only reflects the heat lost from the combined return loop, but does not provide information on the multiple subordinate loops within the systems.

#### 2.2.2. Subordinate Flow and Return Loops

**Figure 2:**
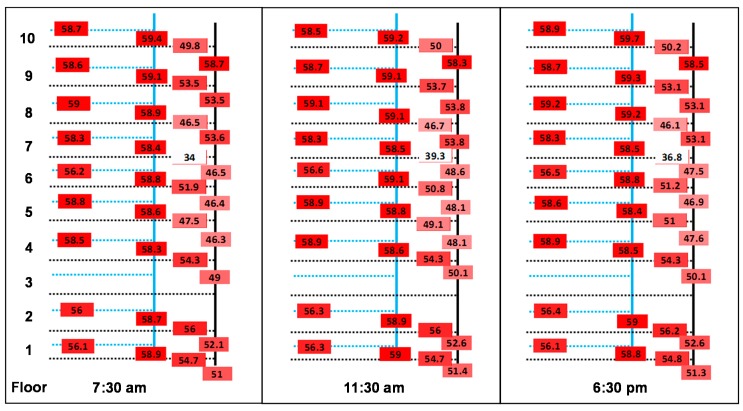
Temperature mapping in System A for principal vertical (full lines) and all horizontal subordinate flow and return loops (dotted lines) on 7 February 2015, at three different times of the day. Blue lines represent hot water, and black lines represent recirculation lines.

Continuous monitoring of each subordinate flow and return loop in System A was conducted over two weeks. The system architecture is such that each subordinate loop supplies water to a floor and returns to the principal recirculation vertical pipe. Figure 2 shows an example of the temperature distribution within System A at three different times within a representative day. No relation between the floor number and the temperature decrease was observed. However, it was noticed that the temperature drop in the horizontal subordinate loop had an impact on the temperature measured in the principal loop proportional to the water volume flowing back into the principal loop. Results from Figure 2 represent a point in time when hot water usage was high. However, similar trends were observed when considering mean temperatures measured over a two-week period (Table S1). Mean hot water temperatures from the principal and subordinate loops were between 55.8 and 59.2 °C with low variability observed throughout the monitoring period (Table S1). Lower temperatures were measured in the horizontal subordinate loop, with temperature losses of up to 2 °C between vertical and horizontal measuring points at a given floor, despite the close proximity of those points (less than 1 m apart). The monitoring of horizontal subordinate return loop temperatures highlighted significant discrepancies between different floors, with mean temperatures varying from 39.8 to 53.1 °C during the monitoring period (Table S1). Lower temperatures were observed during high demand periods, as shown in Figure 2. These results identify sectors where mean hot water temperatures cannot be maintained at the target value of 55 °C.

These results illustrate how defective hydraulics at specific subordinate loops can go undetected if temperature monitoring is only conducted on the principal flow and return loops. As is the case for the seventh floor, water temperatures of well below 55 °C were consistently recorded in the subordinate return loop. As it merges in with the principal return loop flow, the effect is diluted by water of higher temperature returning from other subordinate loops. The factors that are likely to drive important temperature decreases within a subordinate loop are: (1) the presence of hydraulic deficiencies specific to those areas; (2) the presence of cold water intrusion into the hot water recirculating system; and (3) the lack of pipe insulation. A thorough survey of the piping system revealed no insulation deficiencies. However, detailed investigation revealed that the piping system on the seventh floor had several dead legs. Moreover, the piping configuration is such that convective mixing is possible, which may have implications for *Legionella* growth [31].

The detailed investigation also revealed that five rooms were fed through an independent hot water flow and return loop system, with more than 20 m of piping separating the connections of the subordinate horizontal flow and return loops, from the faucets within the rooms. For this specific loop, monitoring revealed an infeed temperature of 49.5 ± 2.7 °C and a return temperature of 34.1 ± 1.5 °C. Minimal water circulation was also recorded at the average speed of 0.03 ± 0.01 m/s, compared to the mean circulation speed in the subordinate horizontal loop of 0.13 ± 0.02 m/s. The water volume contained within this piping (estimated to be 61.6 L) was therefore quasi-stagnant between water usages. In addition, the presence of three dead legs in the proximity of the connection were identified. All of these factors contributed to the inability of the HWS on the seventh floor to maintain adequate temperatures, with a mean heat loss of 18.2 °C occurring between flow and return loops.

A detailed investigation was also conducted on System B, which has a different configuration than System A (Figure 1). In System B, hot water temperatures feeding into the four risers were not significantly different from those in System A, with mean values between 54.8 and 58.3 °C (Table S1). High-risk areas for *L. pneumophila* growth were identified through temperature diagnostics of the subordinate flow and return loops for each vertical riser. The ground and first floors were fed by a horizontal flow and return loop, independent of the four identified risers. The first floor was fed through multiple loops originating from the ground floor subordinate horizontal flow and return loops. Monitoring was conducted on one of these loops in the vicinity of riser 4P. A temperature loss of 12.4 °C was measured between the flow and return horizontal pipes (Table S1), suggesting defective recirculation in the loop. Upon investigation of the piping within this loop, dead legs equivalent to 30 m of linear piping were identified and removed. A defective valve was also preventing recirculation of water in a large section of the loop.

Significant water temperature reduction in a subordinate flow loop can be caused by numerous factors: (1) prolonged stagnation in dead legs or in low usage areas; (2) insufficient insulation [20]; (3) insufficient recirculation flow rate causing increased residence time and thermal losses, especially during periods of low use, such as night-time; (4) the absence of recirculation in specific sectors due to defective equipment or inadequate hydraulic balancing; (5) flow inversion in piping due to pressure variation during high demand periods; and (6) mixing of cold water with the hot water system through defective valves on faulty taps. The results obtained from temperature diagnostics can be used to identify problem areas and to determine the cause of the temperature decrease, thus allowing remediating action to be taken.

#### 2.2.3. Tertiary Loop and Points-of-Use Characterization

**Figure 3:**
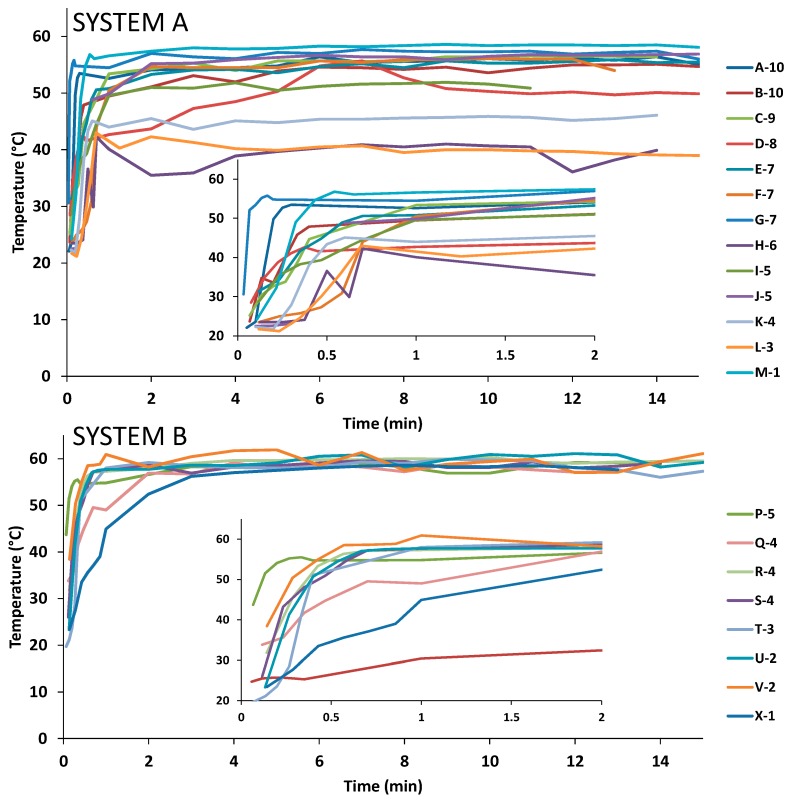
Individual temperature profiles for points-of-use in Systems A and B, with the insert graph zooming in on the first 2 min. The legend indicates the tap as listed in Table S2 with the floor number.

Complete characterization of the sampled faucets was also conducted and is presented in the Supplementary Material (Table S2). As part of the risk classification approach, the measurement of temperatures at the point-of-use provides information on the maximum temperature reached and the time taken to reach this temperature. A temperature of 55 °C should be reached at all taps within 1 to 2 min. Temperature profiles were generated on hot water from 13 taps in System A and eight taps in System B, to evaluate their capacity to deliver appropriate temperatures. Mean temperatures observed on first flush, after 1 and 2 min of flow and the maximum temperature reached at the tap are summarized in Table 2. Detailed temperature profiles are presented in Figure 3.

The analysis of temperature profiles revealed that points of utilization located on floors identified as deficient took longer to reach their maximum temperatures (Figure 3). In System A, two mixer faucets recorded temperatures of around 40 °C. These two data points lowered the percentage of taps reaching 55 °C; however, even after excluding these taps from analysis, the percentage of taps reaching 55 °C after 2 min of flow was still below 50%. An issue was also highlighted with the tap located on the eight floor, in System A (Figure 3). This was the only tap that required more than 5 min to reach its maximum temperature and that could not maintain it, over the duration of the profiling. This tap was located in close proximity to where the second case of legionellosis occurred. Despite the low temperature observed in the subordinate return loop, temperature profiles show the capacity of the system as a whole to draw hot water at the required temperature. This suggests that the low temperatures observed in the return loop are caused by a lack of recirculation and a stagnation of the water at times of low hot water usage.

The absence of residual chlorine in all hot water samples was observed in this study. In addition, the dissolved copper concentration was measured in hot and cold water from a selected number of taps. This enabled quantification of the contribution of the copper-silver ionization system compared to that of the copper piping. Sampling was performed after 15 min of flushing and after a controlled stagnation of 30 min. The value obtained after flushing is an indicator of the concentration of ions provided by the copper-silver ionization system, and the value obtained after stagnation is representative of the ions contributed by the copper piping and components. Results are presented in Table 3. The target concentration for the copper silver ionization system was 400 µg/L of dissolved copper. Results show the failure of both systems to meet the target concentration. The generation of copper-silver ions was driven by a feedback loop system, using the measured copper concentration in the main recirculating pipe prior to the ionization system as the reference value. The value measured in this location does not account for the cold water added into the system to replenish consumed water through hot water usage. In the case of System A, the copper concentrations were closer to the desired level, suggesting that the copper-silver ionization system was capable of providing the desired concentration at the point of copper measurement, but this concentration was insufficient to offset the addition of untreated cold water into the system. Relocating the copper measurement point after the cold water addition would provide a more accurate dosage of ions into the system. Incoming municipal water was a small contributor for copper concentration in the system water, with 80 µg/L measured in System A and 108 µg/L measured in System B, whereas the silver concentration was below the detection limit.

Results collected after stagnation indicate an important contribution from the copper piping and other components, to the measured copper concentration. This contribution is also observed in cold water, where the copper concentration increases by more than two-fold after stagnation. The contribution of the connecting pipe from the copper piping to the tap can also be observed when comparing the results of taps connected with copper *versus* flex piping. In cold water, the dissolved copper concentration was significantly higher (50%, *p* < 0.05) in copper-connected taps after stagnation compared to taps connected with flexible hoses. Results also evidence the low concentrations of silver present in water. According to recommendations, the silver ion concentration should represent 10% of the copper concentration. Based on these results, the copper-silver ionization systems were only partially effective at disinfecting water Systems A and B.

This raises questions with regards to the ability of copper-silver ionization to control levels of *L. pneumophila* in hot water systems, in certain conditions. Although copper-silver ionization has been used successfully in hospital water systems [23], its ability to maintain disinfection efficiency over time has been questioned, especially in the absence of other factors limiting *L. pneumophila* growth, such as adequate thermal control. A recent outbreak was reported in a hospital using copper-silver ionization as their principal disinfection method [3]. Mean ion concentrations were of 0.3 mg/L for copper and 0.02 mg/L for silver; values at the lower end of the scale of recommended applicable concentrations [23]. The authors highlight the low levels or total absence of residual chlorine at the time of the outbreak, likely associated with an increase in organic matter in the water system due to construction work. They also report the failure of 0.4 mg/L of copper combined with 0.04 mg/L of silver ions to reduce the viability of *Legionella* in a 24-h laboratory test [3]. In addition, systems with elevated concentrations of copper were reported in the plumbing of premises lacking a copper-silver ionization system [32].

### 2.3. Legionella pneumophila Occurrence in the Hot Water Systems

Following initial detection of *L. pneumophila* before and after thermal disinfection, an ongoing plan was developed to monitor the evolution of *L. pneumophila* concentrations in the hot water systems. The occurrence was evaluated by culture and qPCR. The percentage positivity did not significantly change through the year following thermal disinfection and the implementation of a thermal regime (Table 4). The significance of the culture method limit of detection is highlighted in the present analysis. In this case, initial evaluation before and after thermal disinfection revealed a percentage positivity that decreased from 85% to 0%, in System A. Follow-up measurements indicated 20% to 45% positivity in System A. This can be attributed to the high limit of detection in the culture method used before and after sampling. For the ongoing monitoring, a different laboratory was used, and the detection limit improved to 10 CFU/L. As a result, the percentage positivity increased to 33% in culture. It should be noted that all samples, except one, were below the detection limit in the initial sampling (1000 CFU/L). The limit of detection is a critical parameter when evaluating the efficiency of disinfection or corrective measures. Culture method detection limits should be improved through increased pre-treatment and filtration of larger volumes of water.

**Figure 4:**
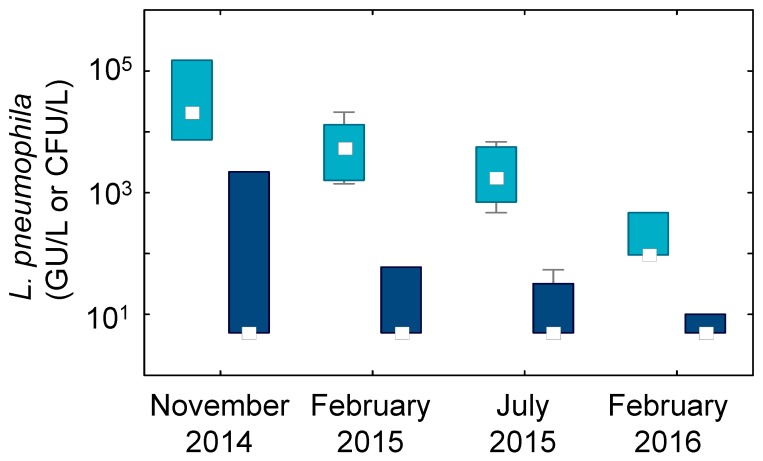
Levels of *L. pneumophila* in System A by qPCR (light blue) and culture (dark blue) as monitored over time. □ = median; boxes = 10%–90%; whiskers = min-max; *n* = 10.

The percentage positivity significantly decreased throughout the monitoring period in System A and was below the recommended 30% positivity [33] for the last two sampling dates. Furthermore, the level of contamination measured by qPCR and culture has steadily decreased over the year following the application of the thermal regime and corrective measures. Figure 4 presents the levels of *L. pneumophila* in System A, measured during each sampling campaign.

As shown in Figure 4, qPCR results were significantly lower in the samples from February 2016 compared to the previous dates. A reduction of culturable levels is also observed over time in System A, but results were not significantly different when tested with the Kruskal–Wallis test. Nevertheless, levels of culturable *L. pneumophila* were significantly higher prior to thermal disinfection in August 2014 (*p* < 0.001) compared to levels measured over the following 18 months (Table 1, Figure 4).

Despite the reduction in levels measured by culture and by qPCR in the first year, an elevated percentage of contamination remained by qPCR. As such, an investigation of the central water heating unit was initiated in May 2015. The objective was to identify the presence of a potential reservoir promoting the proliferation of *L. pneumophila*, despite the implementation of corrective measures and the maintenance of appropriate temperatures. Sampling of water in the pre-heating unit preceding the water heater revealed contamination of the device with the outbreak strain [34]. It was hypothesized that the heat exchanger acted as an amplification unit for *L. pneumophila*, especially considering the piping system, which allows recirculation water to flow through the heat exchanger, as seen in the piping schematic in Figure 1. The combination of the *L. pneumophila* load in the water returning to the water heater and the favorable growth conditions present in the heat exchanger (surface, temperatures and stagnation) promoted proliferation and led to high-level contamination in hydraulically-deficient areas. The inability to maintain adequate temperatures in those sectors enabled *L. pneumophila* growth.

**Figure 5:**
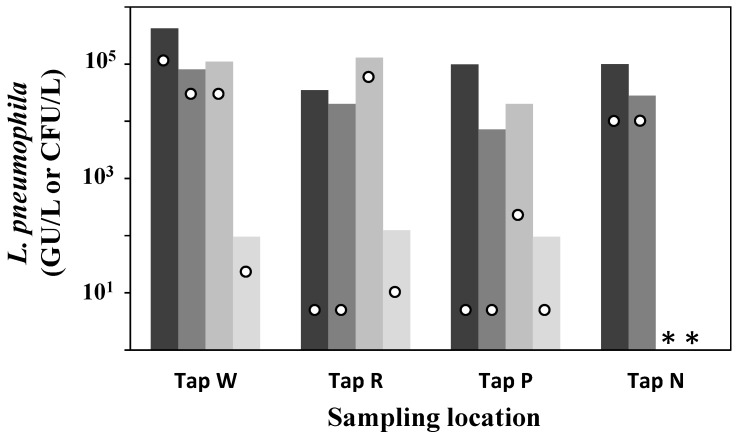
Levels of *L. pneumophila* in System B by qPCR (bars) and culture (circles) measured at three different points over time: November 2014 (black), February 2015 (dark grey), July 2015 (grey) and February 2016 (light grey). Results below the limit of detection (LD) were represented as 0.5LD; LD = 10 CFU/L and 190 genomic units/L (GU/L). Tap N was not sampled in July 2015 and February 2016 (*) due to the presence of point-of-use filtration at the tap.

In System B, the percentage positivity by culture and qPCR was above 30%, except for the last sampling, where qPCR results were all below the detection limit (Table 4). Based on the taps’ positivity, System B is still considered at risk. A small subset of four points-of-use was monitored over time. Sampling points were selected in areas of high risk for *L. pneumophila*, according to the results from the diagnostic tool. A significant decrease was observed by qPCR on the last sampling date, but no clear trends were observed in culture (Figure 5). The removal of a total of 30 m of dead leg pipes and the replacement of a deficient valve were done in the ground floor loop feeding tap W. Despite the less efficient thermal disinfection in System B (Section 2.1), the implementation of thermal regime and corrective measures to eliminate hydraulic deficiencies can lead to a reduction of the *L. pneumophila* contamination. Furthermore, the qPCR results suggest a decline in the total population size.

These results suggest that the effectiveness of thermal disinfection is dependent on the established hydraulic conditions and conditions favorable to *Legionella* growth. In areas where temperature control is insufficient, distal amplification may occur, resulting in a number of taps remaining positive for *Legionella* [8,25,35]. This was also evidenced in the present study, where in July 2015, additional sampling from one of the rooms on the seventh floor with deficient recirculation revealed an *L. pneumophila* concentration of 1200 CFU/L in culture. Therefore, maintenance of the thermal regime is an important driver in maintaining the control of distal amplification of *L. pneumophila*, near the points-of-use. However, this is difficult to achieve in older buildings that have an unbalanced distribution of water flow and temperature, due to a multitude of system changes over time (renovations, changes in water usage, building additions, new equipment).

The temperature diagnostic approach identified areas where hot water temperatures could not be maintained at or above 55 °C. These areas provide more favorable conditions for *L. pneumophila* growth and therefore increase the risk of exposure. Culture and qPCR detection confirmed the presence of *L. pneumophila* in areas where temperatures could not be maintained. However, mean copper levels above 400 µg/L after stagnation at the point-of-use did not significantly affect the presence of *L. pneumophila* in the water.

## 3. Experimental Section

The investigation was conducted in a 400-bed university hospital in Sherbrooke, Canada, on two separate hot water systems feeding Wing A (300 beds) and Wing B (100 beds). Both systems are supplied by municipal chlorinated surface water. The average pH was 7.5 (min 7.1; max 8.0; *n* = 363). A detailed characterization of the hot water systems was conducted as part of the investigation and is presented in the Results Section. Two nosocomial cases of *L. pneumophila* infection were reported in August 2014 in Wing A, from the oncology ward (7th floor) and from the surgery ward (8th floor). Following the reported cases, 250 mL of first flush hot water were collected from 25 taps (bath and sink) in the outbreak wing (System A) and 9 taps associated with System B. These samples were sent to an outside accredited laboratory for analysis. Results from this sampling were presented as the “before thermal disinfection” dataset in Table 1. The limit of detection was 1000 CFU/L.

### 3.1. Thermal Disinfection

A complete thermal disinfection of both systems was conducted as follows: temperature in the hot water system was raised over 70 °C and maintained for 60 min, before flushing at individual points-of-use. Water was discharged from each point-of-use for at least 7 min with hot water at temperatures over 70 °C. All of System A was disinfected over a single intervention of 14 h, whereas System B was disinfected over two interventions. The large number of points-of-use in System B made it impractical to perform thermal shock disinfection in a single intervention. First, the risers furthest from the water heater (4P and 11J) were thermally disinfected. After the first intervention and until the second intervention was completed, recirculation water from this sector was flushed directly down the drain. The second intervention was performed one week later. Immediately after thermal disinfection, resampling was conducted on 18 points in System A and 9 points in System B. Results from this sampling effort were referred to as “after thermal disinfection” in Table 1. The following corrective measures were also implemented after the thermal disinfection: the hot water temperature set point was increased to 60 °C, prior to its distribution around the hospital; all points-of-use in both wings were rinsed, weekly with hot and cold water for 2 to 3 min and monthly with hot water only; the temperature in the principal recirculation loop was monitored, ensuring it remained at or above 55 °C.

### 3.2. Hot Water Recirculation Loop Diagnostic

Continuous temperature monitoring was performed over a period of 2 weeks in System A and two successive periods of 2 weeks in System B, due to the higher number of loops that required monitoring. In System B, the monitoring was carried out on the floors and columns deemed most at risk for the proliferation of *Legionella.* The monitoring point locations are shown in Figure 1. The temperature of water was assessed using self-adhesive sensors, SA1XL and RDXL4SD Datalogger (Omega, Laval, QC, Canada), installed on horizontal and vertical pipes for hot water and recirculation, on monitored floors (Figure 1). Temperatures were recorded every 5 min during the monitoring period. The hot water temperature of the principal flow and return loops was recorded by the hospital building management system every 5 min, at points immediately after the water heater in System B and after the recirculation valve in System A. The recirculation valve has a set point of 60 °C and uses recirculation water or municipal cold water to lower the water temperature coming out of the water heater (70 °C). The principal recirculated water temperature was measured at a point before the recirculation pump, in both systems.

### 3.3. Points-of-Use Characterization

Ongoing monitoring of water quality following the implementation of corrective measures was conducted four times: November 2014, February and July 2015 and February 2016. Sampling points were selected from the cold water system, the hot recirculation loop and points-of-use in each wing. A description of points sampled in each campaign is presented in Table S1. System points were flushed for 2 min prior to collecting 2.25 L of water. For points-of-use, 2.25 L of hot water were collected immediately upon opening the tap (first flush). Water was sampled in sterile polypropylene bottles with sodium thiosulfate (final concentration of 1.1 mg/L). Microbiological analyses were conducted as described in Section 3.4.

Points-of-use were characterized for their temperature, residual chlorine and copper/silver content. Temperature profiles were established for each point-of-use over a period of 15 min using the digital thermometer VWR Traceable^®^ (VWR International, Edmonton, AB, Canada) inserted into the sampled water. Temperature was measured in 500-mL increments for the first 3 L and every minute until 15 min at a fixed flow rate of 6 L/min. Residual chlorine in all samples was measured on-site using a Pocket Colorimeter™ II (Hach, Loveland, CO, USA). Total copper and silver concentrations were measured in hot water (*n* = 23) and cold water (*n* = 12) at the point-of-use, after 15 min of flushing and 30 min of stagnation, by inductively-coupled plasma mass spectrometry [36] after acid digestion of 0.5% HNO_3_ for 24 h.

### 3.4. Microbiological Analysis

Water samples were mixed thoroughly and divided into equal samples for the isolation and quantification of *Legionella* spp. and *L. pneumophila* by culture and qPCR. The culture of *Legionella* spp. and *L. pneumophila* was performed according to the AFNOR (Association Française of Normalisation: the French Standards Association) NF-T90-431 [37]. Briefly, a small sample volume (0.2 mL) and additional volumes filtered through sterile 0.4-µm polycarbonate membranes (100 mL, 10 mL and 1 mL) (Maine Manufacturing, LLC, Sanford, ME, USA) were plated on glycine, vancomycin, polymyxin and cycloheximide (GVPC) selective agar (Innovation Diagnostics Inc., Blainville, QC, Canada) and incubated at 36 °C for 10 days. Filtered samples were subjected to acid treatment (pH = 2; 5 min) prior to plating. Typical colonies that developed after 4 to 10 days were sub-cultured on confirmation plates (buffered charcoal yeast extract (BCYE) with/without cysteine or blood agar) for 2 to 4 days, at 36 °C. Resulting colonies that developed on BCYE agar, but neither on blood agar, nor on BCYE without cysteine, were considered as *Legionella* spp. Confirmation of *L. pneumophila* was conducted using the *Legionella* latex test (DR0800, OXOID Limited, Nepean, ON, Canada). The calculated detection limit for the culture method was 10 CFU/L for both *Legionella* spp. and *L. pneumophila*. Detection by qPCR was performed on 1 L of sampled water, following the AFNOR NF-T90-71 method [38] with the GeneDisc^®^ Cycler, *Legionella* DUO GeneDisc^®^ (Pall). This plate allows duplicate analysis of five DNAs extracted from water samples and a negative control. An internal control for the inhibition of *L. pneumophila* and *Legionella* spp. is also included. The primers and probes used are specified by the AFNOR NF-T90-471. All culture and qPCR analyses were performed by the Centre d’Expertise en Analyse Environnementale du Québec (CEAEQ).

### 3.5. Statistics

Culture and qPCR results for Systems A and B were determined to be non-parametric. The Kruskal–Wallis test was used to determine if there was a significant change in culture and qPCR results, in each system over the four different sampling dates. Results were considered significant if *p* ≤ 0.5.

## 4. Conclusions

In this study, thermal disinfection of one hot water distribution system implicated in previous cases of legionellosis resulted in a significant decline in culturable *L. pneumophila* concentrations. However, limited success was observed in a second hot water system at the same hospital. Simple and low cost temperature monitoring was used to investigate the hydraulic regime in the water system of the outbreak wing. Results revealed significant recirculation failures that were most likely the cause of the outbreak. The combination of an enhanced thermal regime with the implementation of corrective measures (flushing taps weekly, removing dead legs, repairing faulty devices) to ensure the maintenance of this regime further helped to reduce the levels of *L. pneumophila* in the outbreak wing, as measured by qPCR and culture. The percentage positive culture was reduced below 30%, and a significant reduction in the level of contamination in the system was observed by qPCR. These observations emphasize the importance of rigorously maintaining the thermal regime through local interventions, to minimize *L. pneumophila* concentrations at the points-of-use. Where important systematic hydraulic deficiencies were confirmed, there was no significant reduction in *L. pneumophila* at the points-of-use until they were addressed, regardless of lower concentrations in the primary and secondary loops. Quantitative PCR-based monitoring can complement culture-based methods and has proven useful at monitoring long-term changes in *L. pneumophila* levels in the presence of disinfecting agents affecting cell culturability. The results imply that local conditions may drive the risk of legionellosis in older buildings and that monitoring of the conditions in the primary distribution system should not be relied upon to evaluate risk. The temperature diagnostic method applied on the subordinate flow and return loops was a useful tool to identify specific areas with deficient equipment or hydraulics that prevented the maintenance of temperatures over time. As such, it allowed corrective measures to be prioritized in those areas of most need, thus reducing the risk of patient exposure to *L. pneumophila*.

## Figures and Tables

**Table 1:** Mean level of *L. pneumophila* in positive samples before and after thermal disinfection.

*L. pneumophila* Positivity	System A		System B	
	Before	After	Before	After
Mean *L. pneumophila* levels ^1^ (CFU/L)	27,200	<LD ^2^	1700	2000
Number of samples	27	20	10	10
% positives	81.5	0	50	40

^1^ Calculation only included positive samples; ^2^ LD = limit of detection, 1000 CFU/L.

**Table 2:** Mean hot water temperature measured at the tap in Systems A and B and the percent of taps reaching 55 °C after various flow times.

	*n*	Mean Temperature ± SD (°C)				% at 55 °C after 2 min	% T_MAX_ ≥ 55 °C
		First Flush	1 min	2 min	Max		
System A	13	25.2 ± 3.4	48.8 ± 5.4	50.4 ± 6.6	52.5 ± 6.1	31%	54%
System B	8	30.0 ± 8.3	55.0 ± 5.4	57.2 ± 2.1	59.0 ± 1.3	88%	100%

**Table 3:** Mean dissolved copper and silver concentrations in hot and cold water at the tap after 15 min of flushing and 30 min of stagnation for System A and System B.

System	Prior stagnation	Copper (µg/L)		Silver (µg/L)	
		Hot Water *n* = 23	Cold Water *n* = 12	Hot Water *n* = 23	Cold Water *n* = 12
System A	15-min flush	332 ± 38	103 ± 40	0.014 ± 0.004	<LD
	30-min stagnation	399 ± 66	215 ± 65	0.016 ± 0.008	<LD
System B	15-min flush	266 ± 44	45 ± 14	0.01 ± 0.003	<LD
	30-min stagnation	357 ± 75	184 ± 52	0.01 ± 0.003	<LD

LD = limit of detection.

**Table 4:** Evolution of positivity for *L. pneumophila* by culture and qPCR in Systems A and B.

	System A				System B			
	14 November	15 February	15 July	16 February	14 November	15 February	15 July	16 February
*n*	9	11	10	10	4	4	3	3
qPCR	100%	91%	90%	20%	100%	100%	100%	0%
Culture	33%	45%	20%	20%	50%	50%	100%	67%

## Supplementary Materials

The following are available online at http://www.mdpi.com/2076-0817/5/2/35/s1, Figure S1: Temperature measured at the water heater outlet and on the return loop prior to the water heater for systems A and B, Table S1: Mean temperature measured on horizontal and vertical pipes monitored for hot water and recirculation, Table S2: Faucet characterization and sampling plan.
