# Supplementary material for: Combination of Heat Shock and Enhanced Thermal Regime to Control the Growth of a Persistent Legionella pneumophila Strain
Source: Pathogens. 2016 Apr 15;5(2):35. doi: 10.3390/pathogens5020035 (PMC4931386; doi:10.3390/pathogens5020035)
Supplement: Supplementary file 1 [file pathogens-05-00035-s001.pdf]

Supplementary Materials: Combination of Heat Shock and Enhanced Thermal Regime to Control the Growth of a Persistent *Legionella pneumophila* Strain

Emilie Bédard, Inès Boppe, Serge Kouamé, Philippe Martin, Linda Pinsonneault, Louis Valiquette, Jules Racine and Michèle Prévost

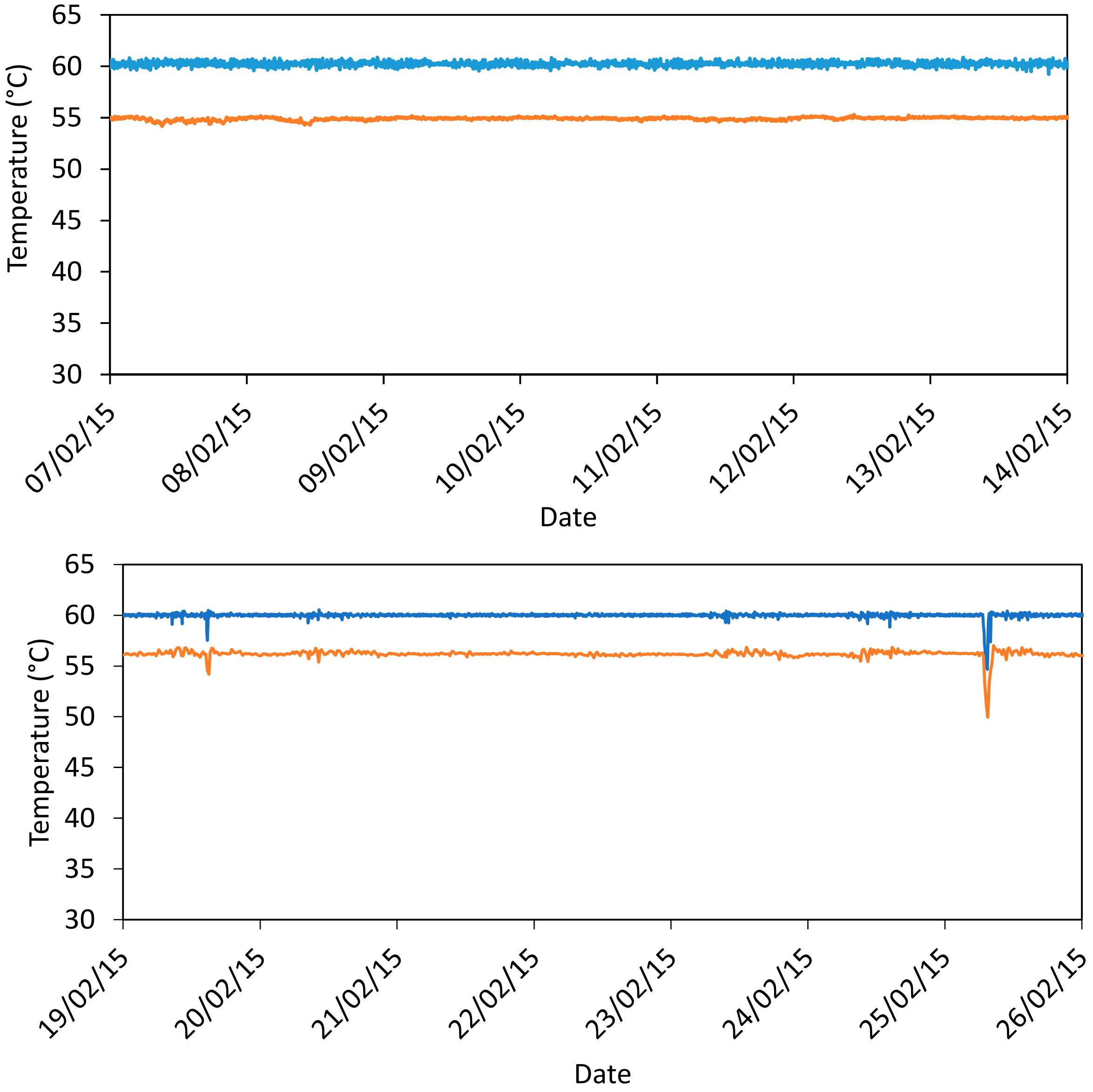

Figure S1. Temperature measured at the water heater outlet (blue) and on the return loop prior to the water heater (orange) for systems A and B.

Table S1. Mean temperature measured on horizontal and vertical pipes monitored for hot water and recirculation.

System	Riser	Floor	Duration (d)	Hot Water Temp. (°C) Mean ± SD		Mean Recirc. Temp. (°C) Mean ± SD		Temp. Loss (°C) Horizontal Loop
				Vertical	Horizontal	Vertical	Horizontal	
A	na	2	17	58.7 ± 0.09	55.8 ± 0.21	53 ± 0.50	55.9 ± 0.36	0
		3	30	57.1 ± 0.28	57.4 ± 0.12	48.8 ± 0.55	54.8 ± 0.14	2.6
		4	17	58.4 ± 0.08	58.7 ± 0.09	50.7 ± 0.78	52.4 ± 1.06	6.3
		5	17	58.5 ± 0.09	58.6 ± 0.09	48.6 ± 0.91	52.5 ± 1.81	6.1
		6	17	58.8 ± 0.08	56.3 ± 0.08	47.7 ± 0.99	50.9 ± 0.79	5.4
		7	17	58.3 ± 0.09	58.2 ± 0.26	48.2 ± 1.34	39.8 ± 4.12	18.4
		8	17	58.9 ± 0.09	58.9 ±0.10	52.0 ± 0.97	44.9 ± 1.02	14.0
		9	17	59.0 ± 0.11	58.4 ± 0.15	53.0 ± 0.55	53.1 ± 0.54	5.3
		10	17	59.2 ± 0.18	58.5 ± 0.57	58.3 ± 0.16	50.1 ± 0.57	8.4
B	12N	2	24	54.8 ± 2.56	57.4 ± 2.71	54.2 ± 2.46	51.9 ± 2.55	5.5
		3	24	55.1 ± 2.58	58.5 ± 2.87	54.7 ± 2.58	55.5 ± 2.60	3
	11R	2	24	58.3 ± 2.76	57.6 ± 2.72	49.5 ± 2.05	53.4 ± 2.42	4.2
		3	24	-	56.2 ± 2.65	-	53.5 ± 2.47	2.7
	11J	3	24	57.5± 2.77	57.3 ± 2.75	53.6 ± 2.45	55.8 ± 2.60	1.5
		4	24	57.9 ± 2.78	58.0 ± 2.79	50.3 ± 2.51	55.5 ± 2.56	2.5
		5	24	-	58.1 ± 2.80	-	55.3 ± 2.56	2.8
		6	24	-	57.9 ± 2.72	-	54.4 ± 2.64	3.5
	4P	2	24	-	56.5 ± 2.70	-	53.2 ± 2.41	3.3
		5	24	55.5 ± 2.58	57.2 ± 2.67	51.4 ± 2.27	51.1 ± 2.69	6.1
		6	24	55.4 ± 2.60	56.8 ± 2.67	43.1 ± 1.59	52.5 ± 2.34	4.3
	SS & 1		3	56.9 ± 0.96	56.5 ± 1.38	53.6 ± 1.53	44.1 ± 1.04	12.4

Table S2. Faucet characterization and sampling plan.

System	Riser	Floor	ID	Type of Faucet	Connection Material	Mitigated	Sampling			
							T° Profiles	Cu-Cold Water	Cu-Hot Water	*Lp*
A	n.a.	10	A	Manual	Flexible piping		x		x	x
		10	B	Manual	Flexible piping		x	x	x	x
		9	C	Foot-operated	Copper		x	x	x	x
		8	D	Manual	Flexible piping	x	x		x	x
		7	E	Manual	Copper		x	x	x	x
		7	F	Manual	Flexible piping		x	x	x	x
		7	G	Manual	Copper		x	x	x	
		6	H	Electronic	Flexible piping	x	x		x	x
		5	I	Electronic	Flexible piping	x	x	x	x	x
		5	J	Manual	Flexible piping		x	x	x	x
		4	K	Electronic	Flexible piping	x	x		x	
		3	L	Electronic	Flexible piping	x	x		x	x
		1	M	Manual	Flexible piping		x		x	
B	6	4P	N	Electronic	Flexible piping	x			x	x
	6	11J	O	Manual	Flexible piping				x	
	5	11J	P	Foot operated	Flexible piping		x		x	
	4	11J	Q	Manual	Copper		x	x	x	x
	4	4P	R	Manual	Flexible piping		x	x	x	
	4	4P	S	Manual	Flexible piping		x		x	x
	3	11R	T	Manual	-		x		x	
	2	11R	U	Manual	Flexible piping		x	x	x	
	2	12N	V	Manual	Flexible piping		x	x	x	
	1	-	W	Manual	Copper		x	x	x	
	SS	-	X	Manual	Flexible piping		x		x	x
